# The enzymes of human diphosphoinositol polyphosphate metabolism

**DOI:** 10.1111/febs.12575

**Published:** 2013-11-05

**Authors:** Mark P Thomas, Barry V L Potter

**Affiliations:** Department of Pharmacy & Pharmacology, University of BathUK

**Keywords:** diphosphoinositol polyphosphate phosphohydrolase, inositol hexakisphosphate and diphosphoinositol-pentakisphosphate kinase, inositol hexakisphosphate kinase, inositol pyrophosphate

## Abstract

Diphospho-*myo*-inositol polyphosphates have many roles to play, including roles in apoptosis, vesicle trafficking, the response of cells to stress, the regulation of telomere length and DNA damage repair, and inhibition of the cyclin-dependent kinase Pho85 system that monitors phosphate levels. This review focuses on the three classes of enzymes involved in the metabolism of these compounds: inositol hexakisphosphate kinases, inositol hexakisphosphate and diphosphoinositol-pentakisphosphate kinases and diphosphoinositol polyphosphate phosphohydrolases. However, these enzymes have roles beyond being mere catalysts, and their interactions with other proteins have cellular consequences. Through their interactions, the three inositol hexakisphosphate kinases have roles in exocytosis, diabetes, the response to infection, and apoptosis. The two inositol hexakisphosphate and diphosphoinositol-pentakisphosphate kinases influence the cellular response to phosphatidylinositol (3,4,5)-trisphosphate and the migration of pleckstrin homology domain-containing proteins to the plasma membrane. The five diphosphoinositol polyphosphate phosphohydrolases interact with ribosomal proteins and transcription factors, as well as proteins involved in membrane trafficking, exocytosis, ubiquitination and the proteasomal degradation of target proteins. Possible directions for future research aiming to determine the roles of these enzymes are highlighted.

## Introduction

Inositol phosphates and inositol polyphosphates are derived from inositol (1,2,3,4,5,6-cyclohexanehexol) and have one or more of the hydroxyl groups phosphorylated. Diphosphoinositol polyphosphates, also known as inositol pyrophosphates, are inositol polyphosphates that have one or more diphosphate groups. The diphosphoinositol polyphosphates were first observed in the early 1990s [1–3]. Subsequently, roles have been ascribed to these compounds with respect to (amongst other things) the regulation of telomere length and DNA damage repair [4,5], inhibition of the cyclin-dependent kinase Pho85 system that monitors phosphate levels [6,7], the response to hyperosmotic and thermal stress [8–10], vesicle trafficking [11,12], apoptosis [13,14] and the regulation of the binding of pleckstrin homology (PH) domains to phospholipids and other proteins [15,16]. The diphosphoinositol polyphosphates have been the subject of several reviews [17–24] that note additional roles for these compounds. All of these reviews, to a greater or lesser extent, mention the enzymes that metabolize these compounds (i.e. two classes of kinase and a family of phosphatases) but only one discusses the enzymes in any detail [18]. As far as we are aware, only one review has been devoted to the enzymes and that concentrates on their structural biology [25]. However, these enzymes have roles beyond being mere biological structures and catalysts and the present study seeks to fill the gap in the literature by reviewing and summarizing these roles.

The most common form of inositol in the human body is *myo*-inositol (*cis*-1,2,3,5-*trans*-4,6-cyclohexanehexol), which is one of nine possible structural isomers of inositol. *Myo*-inositol and its phosphates and polyphosphates have many roles, including regulating ion channel permeability [26,27], phosphate levels [28], transcription, mRNA export and translation [29], insulin signalling, and embryonic development [30]. *Myo*-inositol is also a component of membrane-incorporated phosphatidylinositols [31]. Reviews of the various roles of *myo*-inositol phosphates and polyphosphates continue to be published [32–36]. The diphosphoinositol polyphosphates found in humans are derived from *myo*-inositol. Other forms of inositol occur naturally, although diphosphorylated polyphosphate derivatives of these are not known to occur in humans.

**Fig. 1:**
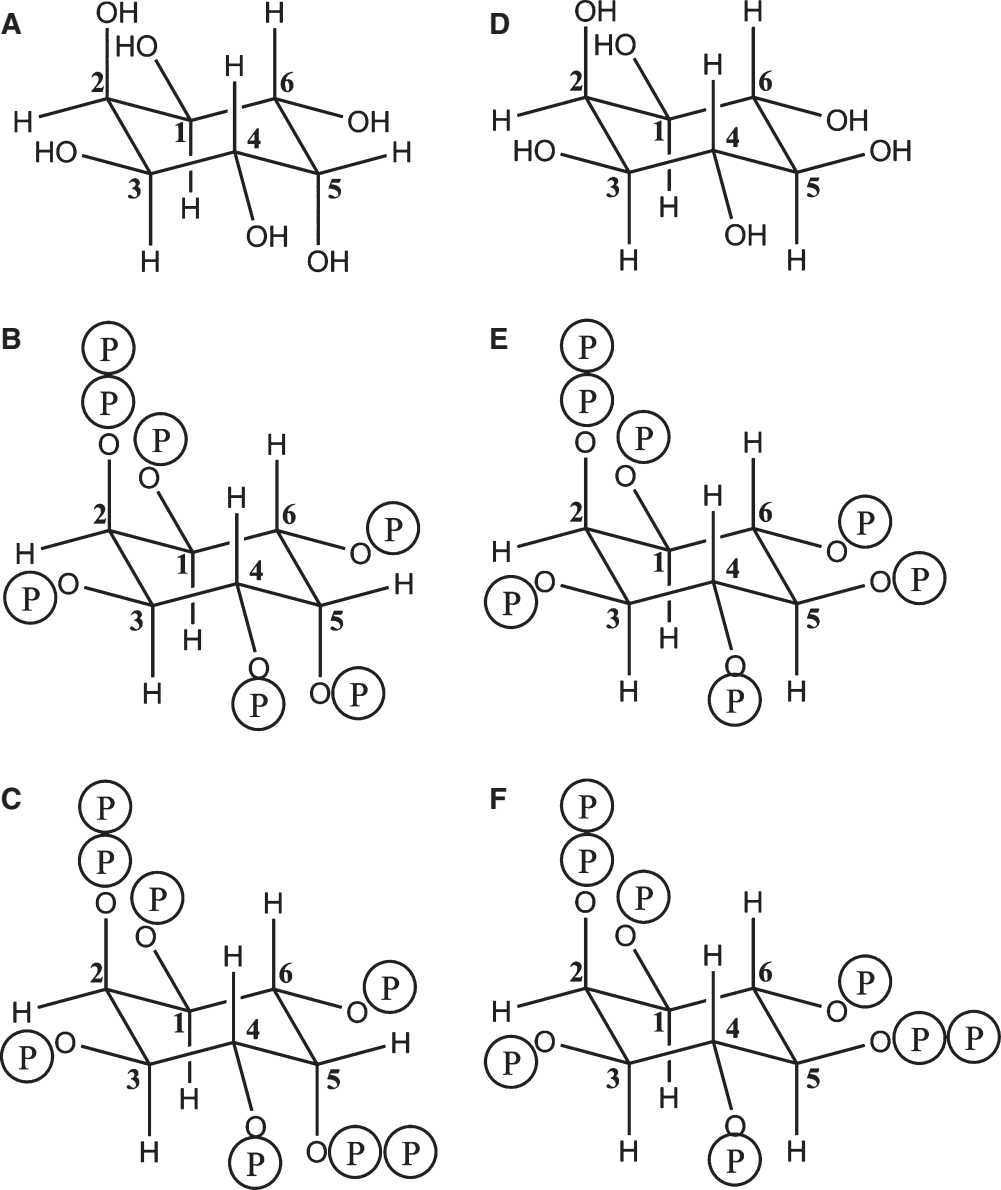
The structures of *myo*- and *neo*-inositol and higher diphosphates. *Neo*-inositol (A), 2-diphospho-*neo*-inositol (1,3,4,5,6)-pentakisphosphate (B) and 2,5-bisdiphospho-*neo*-inositol (1,3,4,6)-tetrakisphosphate (C) are shown, for comparison purposes, alongside their *myo*-inositol equivalents (D–F), the last two of which are not known to occur naturally. The difference between *myo*- and *neo*-inositol lies in the stereochemistry at the 5-position. Prepared in chembiodraw [138].

It should be noted that the diphosphoinositol polyphosphates discussed in the present review and in the reviews cited above are derived from *myo*-inositol and should more properly be called diphospho-*myo*-inositol polyphosphates. Failure to include the ‘*myo*’ in the name is arguably short-sighted because it could lead to confusion with diphosphoinositol polyphosphates of other structural isomers of inositol: for example, 2-diphospho-*neo*-inositol (1,3,4,5,6)-pentakisphosphate and 2,5-bisdiphospho-*neo*-inositol (1,3,4,6)-tetrakisphosphate have both been found in the amoeba *Entamoeba histolytica* [37] (Fig. 1).

**Fig. 2:**
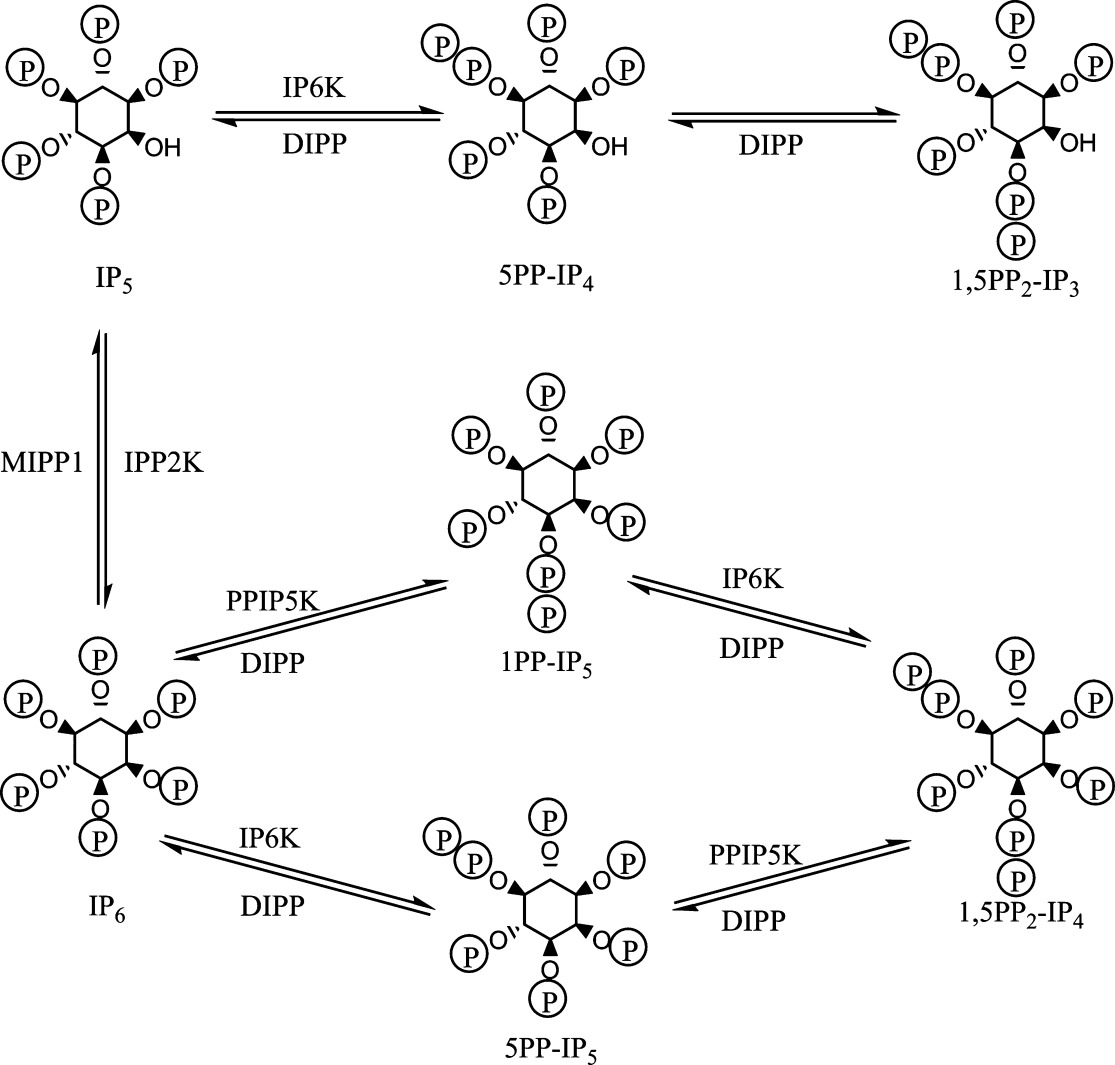
Pathways of diphospho-*myo*-inositol polyphosphate metabolism in humans. 1,5PP_2_-IP_3_ is shown, although this may not be the correct structure (see text). IP_5_, *myo*-inositol (1,3,4,5,6)-pentakisphosphate; 5PP-IP_4_, 5-diphospho-*myo*-inositol (1,3,4,6)-tetrakisphosphate; 1,5PP_2_-IP_3_, 1,5-bisdiphospho-*myo*-inositol (3,4,6)-trisphosphate; IP_6_, *myo*-inositol (1,2,3,4,5,6)-hexakisphosphate; 1PP-IP_5_, d-1-diphospho-*myo*-inositol (2,3,4,5,6)-pentakisphosphate; 5PP-IP_5_, 5-diphospho-*myo*-inositol (1,2,3,4,6)-pentakisphosphate; 1,5PP_2_-IP_4_, d-(1,5)-bisdiphospho-*myo*-inositol (2,3,4,6)-tetrakisphosphate. The enzymes that interconvert IP_5_ and IP_6_ are identified, although they are not discussed in the present review: MIPP1, multiple inositol polyphosphate phosphatase 1 (UniProt ID Q9UNW1); IPP2K, inositol 1,3,4,5,6-pentakisphosphate 2-kinase (UniProt ID Q9H8X2). Figure prepared in chembiodraw [138].

Four diphospho-*myo*-inositol polyphosphates have been found and characterized in humans: 5-diphospho-*myo*-inositol (1,3,4,6)-tetrakisphosphate (5PP-IP_4_), d-1-diphospho-*myo*-inositol (2,3,4,5,6)-pentakisphosphate (1PP-IP_5_), 5-diphospho-*myo*-inositol (1,2,3,4,6)-pentakisphosphate (5PP-IP_5_) and d-(1,5)-bisdiphospho-*myo*-inositol (2,3,4,6)-tetrakisphosphate (1,5PP_2_-IP_4_). How these compounds are interconverted is shown in Fig. 2. Further *myo*-inositol diphosphates and triphosphates generated by human enzymes have been observed *in vitro* but have not been shown to occur *in vivo* [38]. A fifth, uncharacterized diphospho-*myo*-inositol polyphosphate, possibly 1,5-bisdiphospho-*myo*-inositol (3,4,6)-trisphosphate (1,5PP_2_-IP_3_) or a triphosphate, has also been observed *in vitro* and in yeast cells expressing the human enzymes [38]. Other diphospho-*myo*-inositol polyphosphates have been observed in other species [39–41].

The diphosphoinositol polyphosphates undergo rapid turnover [2,42,43]. This turnover is catalyzed by three classes of enzymes: inositol hexakisphosphate kinases (IP6K), inositol hexakisphosphate and diphosphoinositol-pentakisphosphate kinases (PPIP5K), and diphosphoinositol polyphosphate phosphohydrolases (DIPP) (Fig. 2). These enzymes are discussed, in turn, below: unless otherwise stated, it is the human enzyme, or the enzyme in human cells, that is being discussed. It should be noted that the *in vitro* synthesis of diphosphoinositol polyphosphates by inositol polyphosphate multikinase has been reported [44,45]. Because this activity has not been reported *in vivo*, this enzyme will not be discussed herein. Similarly, the *in vitro* hydrolysis of diphosphoinositol polyphosphates by multiple inositol polyphosphate phosphatase has been reported [43] but, because this activity is not known to occur *in vivo*, this enzyme will not be discussed.

The enzymes reviewed herein catalyze reactions that involve phosphoryl transfer. Two other activities of the diphosphoinositol polyphosphates that involve phosphoryl transfer have been observed *in vitro* but not *in vivo*. First, in a reverse of the *in vivo* reaction, diphosphoinositol polyphosphates have been shown to be the phosphate donor in the phosphorylation of ADP to ATP catalyzed by both the rat version of IP6K [46] and the human version of PPIP5K [47,48]. Second, the diphosphates have been shown to act as a phosphate donor in the phosphorylation of proteins. The phosphorylation site is a serine surrounded by several acidic residues [49] and may be a phosphorylation of an already phosphorylated residue: a diphosphorylation [50]. Phosphorylation is inhibited by *myo*-inositol (1,2,3,4,5,6)-hexakisphosphate (IP_6_) [49] but, because the synthesis of IP_6_ is probably compartmentalized [51,52], the phosphorylation of proteins in parts of the cell with low levels of IP_6_ is not precluded.

It should be stressed that this present study comprises a review of the enzymes of diphospho-*myo*-inositol polyphosphate metabolism and not of the diphospho-*myo*-inositol polyphosphates, although these compounds are, necessarily, discussed to some extent. Discussions of the roles of the diphospho-*myo*-inositol polyphosphates are provided in the reviews cited above. It should be further stressed that the present study represents a review of the human enzymes. Where little information is available regarding the human enzymes, the enzymes in other mammalian species (rats and mice) are discussed on the basis that the role of the enzymes in these species is likely to be similar to that in humans. The enzymes in evolutionarily more remote species (e.g. zebrafish and yeast) are discussed only in passing to highlight the differences or make specific points: references to the enzymes in these species and the role of the diphosphoinositol polyphosphates in them may be found in the reviews cited above.

## Inositol hexakisphosphate kinases

The phosphorylation of an already phosphorylated inositol hydroxyl group is catalyzed by enzymes of two different classes. The first class of phosphorylating enzymes to be discovered was the inositol hexakisphosphate kinases (EC 2.7.4.21) of which there are three types [53,54]: type 1 (IP6K1; UniProtKB ID Q92551) is the product of the *IP6K1* gene on human chromosome 3; type 2 (IP6K2; UniProtKB ID Q9UHH9) is the product of the *IP6K2* gene also on human chromosome 3; and type 3 (IP6K3; UniProtKB ID Q96PC2) is the product of the *IP6K3* gene on human chromosome 6. The proteins range in size from 410 to 426 amino acids: a sequence alignment [55] shows that 128 of these residues are conserved across all three proteins, with another 130 residues present in two of the three proteins. The structure of the IP6Ks is not known, although sequence comparisons with other inositol kinases for which 3D structures are known, namely inositol 1,4,5-trisphosphate 3-kinase from human (1W2C, 1W2D, 1W2F [56]; UniProtKB ID P23677) and rat (1TZD [57]; UniProtKB ID P17105), as well as yeast inositol phosphate multikinase 2 (2IEW, 2IF8 [58]; UniProtKB ID P07250) and *Arabidopsis thaliana* inositol phosphate multikinase α (4FRF [59]; UniProtKB ID Q9LY23), all suggest that ATP binds to the C-terminal domain.

Figure 2 shows the reactions catalyzed by the IP6Ks: *myo*-inositol (1,3,4,5,6)-pentakisphosphate (IP_5_) to 5PP-IP_4_, IP_6_ to 5PP-IP_5_ [60] and 1PP-IP_5_ to 1,5PP_2_-IP_4_ [61], with all three reactions being phosphorylations at the 5-position. An ATP molecule serves as the phosphate donor. Measured kinetic parameters for some of these reactions are shown in Table 1.

**Table 1:** Kinetic parameters for the phosphorylation of IP_6_ and IP_5_ by the human inositol hexakisphosphate kinases. All measurements were made with the GST-tagged protein

Enzyme	Substrate	*K*_m_ (μm)	*V*_max_ (μmol·min^−1^·mg^−1^)	Reference
IP6K1	IP_6_	0.6	0.76	[53]
		1.2	0.31	[60]
	IP_5_	6.7	0.26	[60]
IP6K2	IP_6_	3.0	2.0	[53]
		0.43	0.07	[60]
	IP_5_	8.4	0.07	[60]
IP6K3	IP_6_	0.9	0.6	[54]

**Fig. 3:**
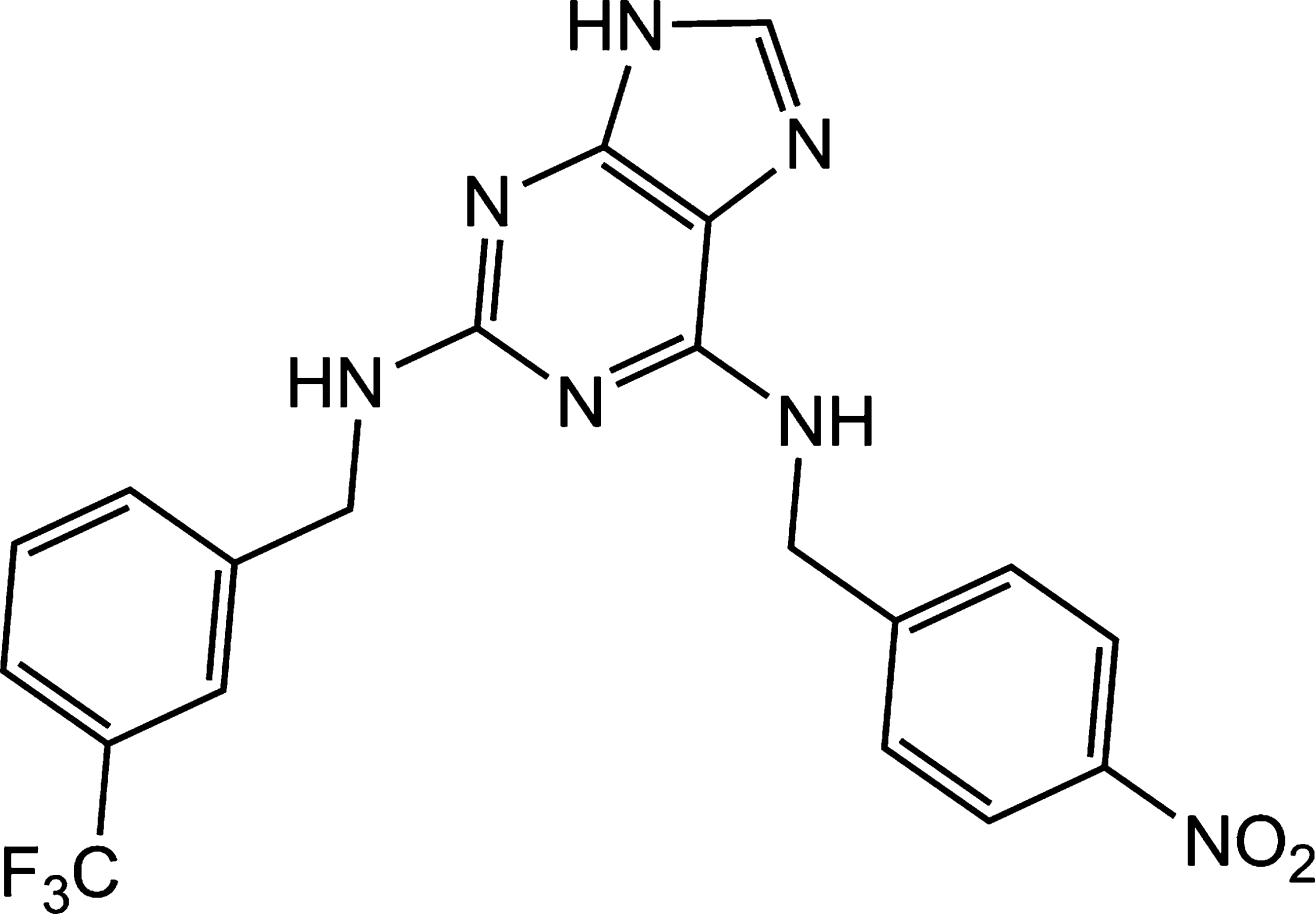
TNP, *N2*-(*m*-(trifluoromethyl)benzyl) *N6*-(*p*-nitrobenzyl)purine, an inhibitor of IP6 kinases [55]. Figure prepared in chembiodraw [138].

An inhibitor, *N*2-(*m*-(trifluoromethyl)benzyl) *N*6-(*p*-nitrobenzyl)purine (TNP) (Fig. 3), has been developed that is a 1000-fold more potent against IP6K than against inositol 1,4,5-trisphosphate 3-kinase, an enzyme considered to be of similar structure based on sequence alignments [56]. This has been used to show that the synthesis of 1,5PP_2_-IP_4_ from IP_6_ occurs predominantly via 5PP-IP_5_ rather than via 1PP-IP_5_ (Fig. 2).

### IP6K1

This enzyme is found in both the cytoplasm and the nucleus [54] and can be phosphorylated at Ser151 [62]. The Human Interactome Database (http://interactome.dfci.harvard.edu/H_sapiens/index.php?page=download) [63–65] identifies IP6K1 as interacting with the exosome component 7 protein (UniProtKB ID Q15024; gene name *EXOSC7*), which is part of a multiprotein complex that degrades RNA [66]. The same interaction is identified in the IntAct database (http://www.ebi.ac.uk/intact/) [67], which also identifies an interaction with the brain calcium channel 1 protein (UniProtKB ID O00555; gene name *CACNA1A*), a voltage-sensitive calcium channel that plays a role in migraine and epilepsy [68]. Most of the work reviewed below has been on the rat (UniProtKB ID Q9ESM0) and mouse (UniProtKB ID Q6PD10) proteins, both of which have amino acid sequences that are highly homologous to that of the human protein.

The Rab3A protein regulates synaptic vesicle exocytosis. The active form of Rab3A has GTP bound; the inactive form has GDP bound. The exchange of GDP and GTP is catalyzed by GRAB, which is a guanine nucleotide exchange factor. In work carried out with rat proteins, it has been shown that IP6K1 binds to GRAB in competition with Rab3A, thus disrupting the regulation of synaptic vesicle exocytosis [69]. An inactive mutant of IP6K1 exerts the same effect, showing that it is the protein–protein interaction, rather than the catalytic activity, that is important for the regulation. IP6K2 does not exert the same effect [69]. This role of IP6K1 in exocytosis has been confirmed in mice in which RNA silencing of IP6K1, but not IP6K2, inhibited insulin secretion in pancreatic β cells [70]. This suggests a role for IP6K1 in diabetes, as also suggested by the finding that, in a human family suffering from type 2 diabetes mellitus, the *IP6K1* gene is disrupted [71]. Reduced insulin levels have also been observed in mice with a version of IP6K1 catalytically inactive as a result of the deletion of the C-terminal ATP-binding domain, although, despite the reduced insulin levels, the mice do not exhibit a diabetic phenotype [72]. This may be explained by the finding that IP6K1 knockout mice have increased sensitivity to insulin as a result of the lack of inhibition of Akt by IP6K1-generated 5PP-IP_5_, which binds to the Akt PH domain [73]. Akt exerts its effect by phosphorylating glycogen synthase kinase 3 (GSK3) on a serine residue in the N-terminal domain: this phosphorylation inhibits GSK3 kinase activity [74]. (GSK3 negatively regulates glycogen synthesis and glucose homeostasis: in type II diabetics, there is increased expression and activity of GSK3 [74].) However, both catalytically active and inactive versions of IP6K1 have also been shown to have a more direct effect on GSK3 catalytic activity by binding to and stimulating GSK3 enzymatic activity in a noncatalytic fashion: IP6K1 binds to the N-terminal region of GSK3 and inhibits the Akt-mediated phosphorylation of GSK3 [75].

The binding of 5PP-IP_5_ to PH domains [in competition with phosphatidylinositol (3,4,5)-trisphosphate, PtdIns(3,4,5)P_3_] disrupts the membrane translocation of PH-domain-containing proteins such as Akt. Mouse neutrophils deficient in IP6K1 have greater levels of membrane-associated Akt than wild-type cells [76]. This results in enhanced NADPH oxidase-mediated production of superoxide, a phenotype replicated in human primary neutrophils with pharmacologically inhibited IP6Ks [76]. Yeast cells lacking Kcs1 (the *Saccharomyces cerevisiae* version of IP6K (UniProtKB ID Q12494) have increased resistance to cell death caused by reactive oxygen species (which cause DNA damage), possibly as a result of activation of DNA repair mechanisms [77]. Hydrogen peroxide inhibits Kcs1 enzyme activity. It has been suggested that this is attributable to chemical modification of cysteine residues because, in murine IP6K1, mutation of Cys221 to alanine or aspartate results in a catalytically dead enzyme [75]. The same study reports that the Cys187 to alanine mutant, although capable of generating an inositol with seven phosphates, is less efficient than the wild-type enzyme at generating an inositol with eight phosphates. By contrast, the mutation of Cys48 or Cys261 to alanine results in greater production of an inositol with eight phosphates than was produced by the wild-type enzyme. Direct equivalents of all these cysteine residues are found in the human protein.

Mice with a version of IP6K1 rendered catalytically inactive as a result of the deletion of the C-terminal ATP-binding domain are deficient in spermiogenesis, which is the final stage of spermatogenesis [72]. Murine embryonic fibroblasts derived from IP6K1 knockout mice can initiate DNA homologous recombination repair but do not complete the process, leading to cell death or the accumulation of chromosomal aberrations [78]. This implies a role for IP6K1 in the maintenance of genetic integrity: in *S. cerevisiae*, the deletion of Kcs1 results in the lengthening of telomeres, whereas the overproduction of 5PP-IP_4_ results in a shortening of telomeres [4,5]. IP6K1 knockout mice have longer blood clotting times as a result of an indirect adverse effect on the accumulation of polyphosphate (containing 60–100 phosphate units), which is necessary for rapid clotting [79]. An intriguing study has reported that, in *S. cerevisiae*, both the kinase domain and the bZIP domain of Kcs1 and the 5PP-IP_4_ product of the reaction catalyzed by Kcs1 are necessary for the proper expression of genes involved in inositol metabolism [80]. Whether this is relevant to the human situation [humans have three inositol hexakisphosphate kinases (with varied subcellular distributions and interactions) as opposed to just the one enzyme in yeast, and Kcs1 is more than twice the size of the human enzymes] is as yet unknown.

### IP6K2

Initial reports suggested that IP6K2 was found only in the nucleus [54] but later reports suggest that it can also be found in the cytoplasm [81,82] and mitochondria (during apoptosis) [14], as well as the nucleus [82].

Several studies, as mentioned below, have suggested a role for IP6K2 in apoptosis. Interferon (IFN)-β suppresses the growth of human ovarian carcinoma xenografts *in vivo* and induces apoptosis of ovarian carcinoma cells *in vitro*. This is the result, at least in part, of a post-transcriptional enhancement of IP6K2 activity by IFN-β [13], possibly through an interaction with phospholipid scramblase 1 (see below). The treatment of cells with cisplatin also causes an increase in IP6K2 activity without increasing the amount of enzyme, possibly suggesting some sort of activating covalent modification of the protein such as phosphorylation [14]. Exposure of cells to IFN-β results in translocation of IP6K2 from the cytoplasm to the nucleus, and inhibition of this translocation inhibits apoptosis [81]. However, translocation to mitochondria associated with the apoptotic process has also been reported [14].

Overexpression of IP6K2 [14,83], accompanied by increased formation of 5PP-IP_5_ and a smaller increase in the amount of an uncharacterized bis-diphosphoinositol polyphosphate [14], increases the sensitivity of some ovarian carcinoma cells to radiation and IFN-β [83] and of multiple cell lines to a range of stressors [14]. It also stimulates the formation of autophagosomes, with the increase being greater in cells challenged with staurosporine than in unchallenged cells [84]. Additionally, there is a reduction in the Akt-catalyzed phosphorylation of mammalian target of rapamycin [84], an enzyme known to play an important role in the formation of autophagosomes [85]. The formation of autophagosomes (consequent upon IP6K2 activation) is associated with the pathogenesis of Huntington's disease [82].

The use of antisense technology to reduce IP6K2 expression results in reduced apoptosis in line with the reduced amount of protein [13,14] and a suppression of autophagosome formation [84]. Using the same method to reduce the expression of IP6K1 and IP6K3 does not reduce apoptosis [14]. However, a mutant IP6K2 devoid of kinase activity reduces apoptosis by 50% [13], suggesting that a protein–protein interaction involving IP6K2 might play a role in the cellular response to IFN-β. One partner in this interaction has been identified as tumour necrosis factor receptor-associated factor (TRAF)2, with the interaction being abolished by mutating the IP6K2 residues Ser347 and Ser359 to alanine [86]. The binding of IP6K2 to TRAF2 interferes with the phosphorylation of transforming growth factor β-activated kinase 1, which leads to the inhibition of nuclear factor-κB signalling. (Abolishing the TRAF2–IP6K2 interaction by mutating Ser347 may reflect a role of phosphorylated Ser347 in the interaction (or in the regulation of the interaction) because this residue has been shown to be phosphorylated by casein kinase 2 [87]).

A heat shock protein, HSP90, has also been identified as binding, through its C-terminal domain, to IP6K2, which results in inhibition of the catalytic activity and a decrease in apoptosis [88]. This inhibition can be overcome by mutation of the HSP90 recognition sequence in IP6K2, by depleting HSP90 through the use of siRNA, or by the use of drugs that bind to the C-terminal domain of HSP90 and inhibit the interaction between the two proteins [88]. Reducing the amount of IP6K2 in the cell abrogates the pro-apoptotic effects of the drugs. Mutation of Trp131 in IP6K2 reduces HSP90 binding, whereas mutation of Arg133 or Arg136 abolishes HSP90 binding.

A third protein shown to bind to IP6K2 is p53 [89]. A cell line deficient in IP6K2 activity was challenged with 5-fluorouracil, a p53-dependent inducer of apoptosis, and sulindac, a p53-independent apoptosis inducer. Those cells challenged with sulindac underwent apoptosis but those challenged with 5-fluorouracil went into G1 arrest. A protein fragment comprising the first 67 residues of IP6K2 competes with the full-length protein for binding to p53, showing that the p53 binding site is located somewhere towards the N-terminus [89].

The binding of TRAF2 to IP6K2 involves residues Ser347 and Ser359 [84]. Residues Trp131, Arg133 and Arg136 are involved in HSP90 binding [88]. The binding site for p53 is somewhere in the N-terminal 67 residues [89]. Although these three parts of the primary sequence could all be in the same area in the three-dimensional structure of the protein, with TRAF2, HSP90 and p53 all binding to the same face of the protein, it may be that different parts of IP6K2 are optimized for binding to different proteins to selectively modify their behaviour or to enable the behaviour of IP6K2 to be selectively modified.

Casein kinase-2 (CK2) is a serine/threonine kinase the expression of which is up-regulated in tumours, resulting in increased cell survival. CK2 inhibitors cause an increase in apoptosis but this effect is not observed in cells deficient in IP6K2. It has been shown that CK2 is able to phosphorylate IP6K2 at Ser347 and Ser356 causing destabilization of IP6K2, thus reducing apoptosis [87].

In addition to the above-mentioned interactions, IP6K2 is identified by the Human Interactome Database (http://interactome.dfci.harvard.edu/H_sapiens/index.php?page=download) [63–65] as interacting with Numb-binding protein 2 (UniProtKB ID Q8N448; gene name *LNX2*), which may be involved in localizing partner proteins to specific subcellular sites [90]. The same database and the IntAct database (http://www.ebi.ac.uk/intact/) [67] identify an interaction between IP6K2 and phospholipid scramblase 1 (UniProtKB ID O15162; gene name *PLSCR1*), which may play a role in (a) the transbilayer migration of phospholipids [91] and (b), as noted above, enhancement of the interferon response [92].

Zebrafish with IP6K2 depleted by antisense technology developed abnormally, with faulty development of craniofacial structures, somites and neural crest cells [93]. This was attributed to a role for IP6K2 in the Hedgehog signalling pathway. By contrast, deletion of IP6K2 in mice resulted in normal embryogenesis, development, growth and fertility [94]. However, these knockout mice were more susceptible than wild-type mice to squamous cell carcinoma in the oral cavity and oesophagus when given water containing a carcinogen. It should be noted that, although the sequence of the mouse protein (UniProtKB ID Q80V72) is highly homologous to that of the human protein, the sequence of the zebrafish protein (UniProtKB ID Q6PBN6) differs quite substantially.

### IP6K3

Little work has been done with this enzyme. It is found in the cytoplasm [54] and can be phosphorylated at Ser242, Thr243 and Ser244 [95]. The mutation of Lys217 to alanine results in a loss of activity, whereas the mutation of Ser325 to alanine results in a large reduction of activity [54], with these two mutations being suggested by the known functions of the equivalent residues in rat inositol 1,4,5-trisphosphate 3-kinase [96] and rat inositol polyphosphate multikinase [44], respectively.

## Inositol hexakisphosphate and diphosphoinositol-pentakisphosphate kinases

The second class of phosphorylating enzymes is the inositol hexakisphosphate and diphosphoinositol-pentakisphosphate kinases (EC 2.7.4.24). In humans, there are two versions of this enzyme [10,97]: type 1 (PPIP5K1; UniProtKB ID Q6PFW1) is the product of the *PPIP5K1* gene on human chromosome 15 and type 2 (PPIP5K2; UniProtKB ID O43314) is the product of the *PPIP5K2* gene on human chromosome 5. Note that some of the older literature on these enzymes names them hsVip based on the fact that they are homologous to the yeast enzyme Vip [97]. The aligned sequences of the two enzymes [97] show that they are highly homologous, having 831 residues in common in total protein lengths of 1433 (PPIP5K1) and 1243 (PPIP5K2) residues. There are no crystal structures of PPIP5K1 but ten of the kinase domain (residues R42-D366) of PPIP5K2: 3T54, 3T7A, 3T99, 3T9A, 3T9B, 3T9C, 3T9D, 3T9E, 3T9F [98] and 4HN2 [47]. The structural biology of PPIP5K2 has been reviewed [25].

PPIP5K1 is widely expressed but with a higher expression in skeletal muscle, heart and brain: it has been reported to be located in the cytosol [10,97] and plasma membrane [16,99]. A proteomics prediction that PPIP5K1 can be found in the nucleus [100] is contradicted by the finding that it is excluded from the nucleus of NIH 3T3 and HEK293 cells [97,99]. Phosphorylations of PPIP5K1 at Ser475, Tyr730, Ser944 and Ser1152 have been reported [101–103]. PPIP5K2 is located in the cytosol [95]. The residues reported to be sites of phosphorylation are: Ser38, Ser504, Ser1006, Ser1016, Ser1172 and Thr1185 [62,101,104–106].

The C-terminal portion of the human protein is a phosphatase-like domain into which a partial PH domain is spliced [99]. The literature reports state that neither the entire protein [10], nor the C-terminal domain [99] has any phosphatase activity towards a number of inositol polyphosphates and diphosphoinositol polyphosphates, although work by another group contradicts this (J. York, personal communication).

Residues P382–E917 of PPIP5K1 form a phosphoinositide binding domain (PBD) that binds PtdIns(3,4,5)P_3_ with *K*_d_ = 96 nm (PPIP5K1) and *K*_d_ =705 nm (PPIP5K2) [98]. Stimulation of PtdIns(3,4,5)P_3_ synthesis in NIH 3T3 cells results in the translocation of PPIP5K1 from the cytoplasm to the plasma membrane [99]. This translocation occurs with just the PBD but not the R399A/R417A PBD double mutant [16], which is able to bind PtdIns(3,4,5)P_3_ only poorly [99]. This suggests that the binding of PtdIns(3,4,5)P_3_ influences the *in vivo* subcellular localization of diphosphoinositol polyphosphate synthesis. The binding of PtdIns(3,4,5)P_3_ is inhibited more strongly by the PPIP5K1 substrates (IP_6_; IC_50_ = 7 μm: 5PP-IP_5_; IC_50_ = 5 μm) than by the products (1PP-IP_5_; IC_50_ = 43 μm: 1,5PP_2_-IP_4_; IC_50_ = 32 μm) [16]. The intracellular concentration of the substrates is greater than or approximately the same as the IC_50_ values but the concentration of the products is far less than the IC_50_ values [25] and so inhibition of PtdIns(3,4,5)P_3_ binding by PPIP5K1 by the products is probably not a factor in enzyme function unless local concentrations of the product are much higher than those measured in the bulk cell; given the high rate of diphosphoinositol polyphosphate turnover [2,42,43] and the restricted rates of diffusion in the vicinity of the plasma membrane [107], such high concentrations may not be impossible. An additional point to consider is that, because the concentration of the substrates is sufficiently high to prevent the PtdIns(3,4,5)P_3_-induced translocation of the enzymes to the plasma membrane, it is possible that this is a mechanism for regulating the cellular response to PtdIns(3,4,5)P_3_: small fluctuations in the concentration of PtdIns(3,4,5)P_3_ might be insufficient to induce the migration of the enzyme, although a sustained stimulus-dependent rise in the PtdIns(3,4,5)P_3_ concentration could induce the movement of the enzyme to the plasma membrane [16]. This argument is strengthened by the finding that the binding of PtdIns(3,4,5)P_3_ to the PH domain of other proteins (GRP1, Akt and SIN1) is inhibited by the PPIP5K substrates, suggesting that PPIP5K1 may play a general role in the regulation of PtdIns(3,4,5)P_3_ signalling cascades [16,108].

Figure 2 shows the reactions catalyzed by the PPIP5Ks: IP_6_ to 1PP-IP_5_, and 5PP-IP_5_ to 1,5PP_2_-IP_4_, with both reactions being the phosphorylation of the phosphate at the inositol 1-position [98]. An ATP molecule serves as the phosphate donor. Measured kinetic parameters for these reactions are shown in Table 2. The disparities between the various reports have been attributed to inter-laboratory variability and differences in the purity of the enzyme and substrate preparations [48]. The phosphorylation of 1PP-IP_5_, IP_5_ and 1,5PP_2_-IP_4_ occurs at negligible rates [10,99].

**Table 2:** Kinetic parameters for the phosphorylation of IP_6_ and 5PP-IP_5_ by the human inositol hexakisphosphate and diphosphoinositol-pentakisphosphate kinases. KD indicates that only the kinase domain of the protein was used in the assay. NR, not reported

Enzyme	Substrate	*K*_m_ (μm)	*V*_max_ (μmol·min^−1^·mg^−1^)	Reference
PPIP5K1	IP_6_	0.12	0.03	[97]
		NR	0.1	[10]
	5PP-IP_5_	0.10	0.13	[97]
		0.34	8.3	[10]
PPIP5K1-KD	IP_6_	0.12	0.42	[97]
	5PP-IP_5_	0.12	1.04	[97]
PPIP5K2	IP_6_	0.13	0.39	[97]
	5PP-IP_5_	0.19	1.38	[97]
PPIP5K2-KD	IP_6_	0.39	43	[48]
	5PP-IP_5_	0.06	190	[48]

**Fig. 4:**
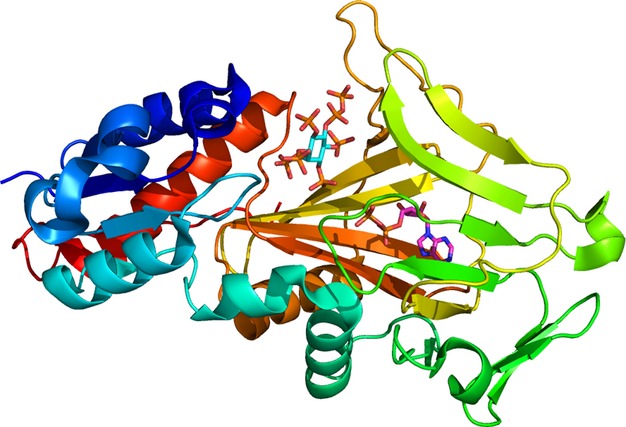
A cartoon showing the structure of the kinase domain of PPIP5K2. The protein, residues R42-D366 constituting the catalytic domain, is coloured blue at the N-terminus through cyan, turquoise, green, yellow and orange to red at the C-terminus. ADP is indicated by purple carbon atoms. 5PP-IP_5_ is indicated by cyan carbon atoms. The αβα domain is shown in blue (residues R42-D124) and red (residues V330-D366). Residues L125-L148 (turquoise) and G244-K329 (yellow and orange) form an antiparallel β-sheet that, together with another antiparallel β-sheet formed by residues P149-D243 (green), forms an ATP-grasp domain. Taken from the 3T9E crystal structure [98]. Image prepared in pymol [139].

**Fig. 5:**
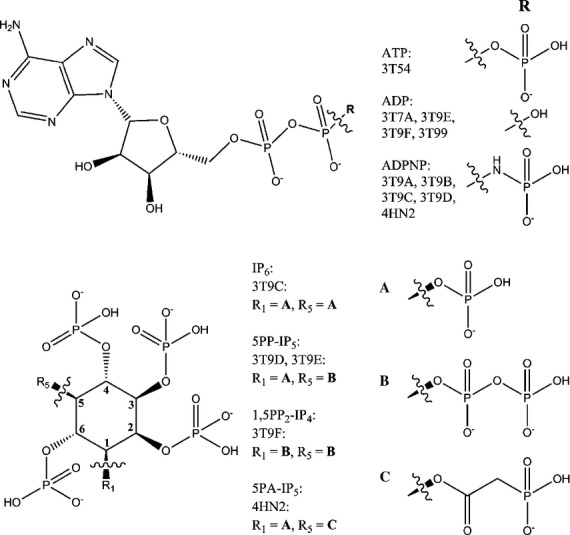
Ligands in the crystal structures of PPIP5K2. Each of the crystal structures contains one of ATP (3T54), ADP (3T7A, 3T9E, 3T9F and 3T99) or ADPNP (3T9A, 3T9B, 3T9C, 3T9D and 4HN2). Five of the structures contain a *myo*-inositol polyphosphate: 3T9C – IP_6_; 3T9D and 3T9E – 5PP-IP_5_; 3T9F – 1,5PP_2_-IP_4_; and 4HN2 – 5PA-IP_5_. Figure prepared in chembiodraw [138].

**Fig. 6:**
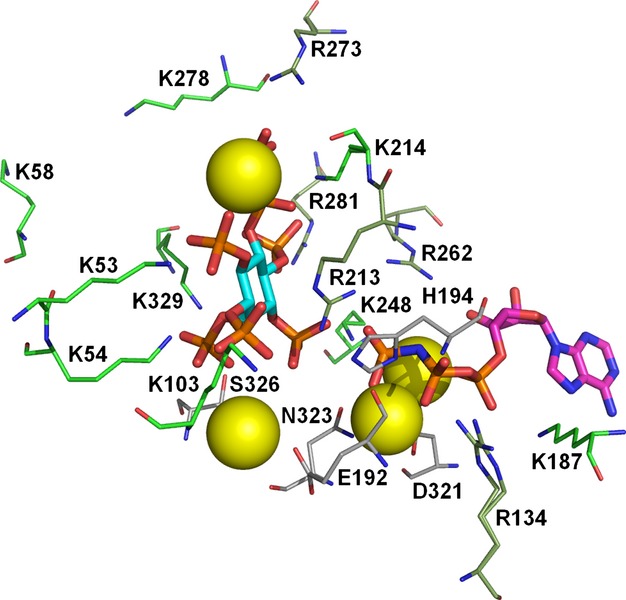
Close-up of the active site of PPIP5K2. 5PP-IP_5_ is indicated by cyan carbon atoms. ADPNP is indicated by purple carbon atoms. Magnesium ions are shown as yellow spheres: their positive charge helps counter the negative charge on the phosphates. With respect to this, they are assisted by the positively-charged side chains of nine lysine residues (light green) and five arginine residues (dark green), one of which (R134) has alternate conformations in the crystal structure. Five other residues (grey) that interact with the magnesium ions or the phosphates are shown. Taken from the 3T9D structure [98]. Image prepared in pymol [139].

The PPIP5K2 kinase domain comprises the N-terminal third of the protein. It consists of an αβα domain and an ATP-grasp domain (Fig. 4). Given the high level of sequence homology, the structure of the PPIP5K1 kinase domain is likely to be similar. All ten crystal structures contain ATP, ADP or ADPNP, and five of them contain an inositol polyphosphate (Fig. 5). The high concentration of negative charge associated with the phosphates on these ligands is accommodated in the binding site by interaction with magnesium ions and a number of lysine and arginine residues (Fig. 6). Mutation of some of these residues results in drops in enzyme activity, with the extent of the drop being dependent on the residue being mutated and the substrate being used [98]. These interactions with the substrate are responsible for the specificity of the enzyme because every phosphate or pyrophosphate in the substrate interacts with at least one of these positively-charged residues or magnesium ions.

The 3T9E structure contains 5PP-IP_5_, ADP and MgF_3_^−^ [98]. This allows insight into the reaction mechanism because the MgF_3_^−^ matches the charge and geometry of the transition state of the phosphoryl transfer reaction [109]. The MgF_3_^−^ has near planar geometry with the magnesium atom positioned between the ADP donor oxygen and the substrate 1-phosphate acceptor oxygen that mimics a trigonal bipyramidal phosphoryl transition state. This suggests an in-line associative reaction mechanism. Dynamics studies suggest that the inositol ring and several side chains move during the phosphoryl transfer reaction [98].

The 4HN2 structure has a diphosphoinositol polyphosphate analogue, 5-phosphonoacetate-*myo-*inositol (1,2,3,4,6)-pentakisphosphate (5PA-IP_5_), bound in the substrate binding site. This ligand inhibits the formation of ATP when the PPIP5K2 kinase domain is incubated with ADP and 1,5PP_2_-IP_4_ with IC_50_ = 129 nm [47]: as noted in the Introduction, the generation of ATP from ADP in a reversal of the normal direction of reaction occurs *in vitro* but is not known to occur *in vivo*. Two other substrate analogues were less potent inhibitors: 5-phosphonoacetate-*myo*-inositol (1,3,4,6)-tetrakisphosphate, IC_50_ = 1386 nm; 2-O-benzyl-5-phosphonoacetate-*myo*-inositol (1,3,4,6)-tetrakisphosphate, IC_50_ = 391 nm. A comparison of the 4HN2 and 3T9D structures (with 5PA-IP_5_ and 5PP-IP_5_, respectively, in the substrate binding site) shows that residues around the 5-position move to accommodate the slightly different structures of the ligand but that the positions of the 1-phosphate and the surrounding residues are similar in the two structures. This similarity may explain the observed phosphorylation of 5PA-IP_5_ at the 1-position [47].

In the IntAct database (http://www.ebi.ac.uk/intact/) [67], an interaction between PPIP5K2 and the Myc protein (transcription factor p64; UniProtKB ID P01106; gene name *MYC*) is identified. This protein is of importance in many cancers [110].

## Diphosphoinositol polyphosphate phosphohydrolases

The dephosphorylation of the diphosphorylated hydroxyl is catalyzed by diphosphoinositol polyphosphate phosphohydrolases (EC 3.6.1.52) of which there are four types: type 1 (DIPP-1; UniProtKB ID O95989) is the product of the *NUDT3* gene on human chromosome 6 [111]; type 2 (DIPP-2; UniProtKB ID Q9NZJ9) (of which there are two isoforms, DIPP-2α and DIPP-2β, produced by alternative splicing) is the product of the *NUDT4* gene on human chromosome 12 [112–114]; type 3α (DIPP-3α; UniProtKB ID Q8NFP7) is the product of the *NUDT10* gene on the human X chromosome [114–116]; and type 3β (DIPP-3β; UniProtKB ID Q96G61) is the product of the *NUDT11* gene, also on the human X chromosome [114–116]. A sequence alignment of the five proteins shows that they have 120 residues in common [116]. DIPP-2β is the largest of the DIPPs, with 181 residues: apart from an insertion of one residue, it is identical in sequence to DIPP-2α. Both DIPP-3 enzymes have 164 residues, with the sequences differing in just one position but both having 146 residues in common with both DIPP-2 sequences. The DIPP-1 sequence (172 residues) differs most from the other sequences, although the differences are largely confined to the C-terminal thirty residues. DIPP-3α is reported to be phosphorylated on S148, T150, S154, S158, S159 and S162 [103]. The significance of this clustering of phosphorylation sites at the C-terminus is unknown.

The relative amounts of mRNA encoding these enzymes have been determined in a range of tissues. The mRNA encoding DIPP-1 is found in the placenta, lung and kidneys and, at higher levels, in the brain, heart, pancreas and liver [111], whereas that encoding DIPP-2 is found in the heart and, at lower levels, in skeletal muscle, the pancreas and kidney, with even weaker expression in the brain, placenta, lung and liver [112,115]. The protein is found in the cytoplasm as are both forms of DIPP-3 [115]. The tissue distribution of mRNA encoding DIPP-3α and DIPP-3β has been more extensively studied [116]. DIPP-3α is found in the brain and liver and, at lower levels, in the testis, prostate, ovary, placenta, spleen, pancreas, kidney, lung and heart, although not in skeletal muscle, the thymus, small intestine, colon or peripheral blood leukocytes. DIPP-3β is expressed in the brain, pancreas and testis and, at lower levels, in the heart, lung, small intestine, thymus, prostate and ovary, although not in the liver, placenta, kidney, spleen, skeletal muscle, colon or peripheral blood leukocytes. However, these results have been partially contradicted (and partially confirmed) by the finding that DIPP-3 is expressed in the testis and brain but not in the uterus, spleen, thymus, small intestine and peripheral blood leukocytes [115].

**Fig. 7:**
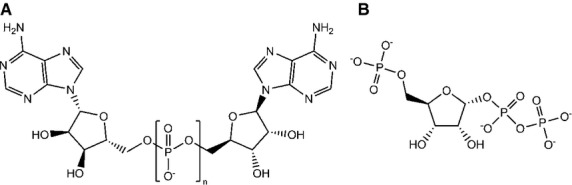
(A) The structure of diadenosine oligophosphate (Ap_n_A). When *n* = 5 (Ap_5_A), the cleavage products are AMP and p_4_A. When *n* = 6 (Ap_6_A), the cleavage products are AMP and p_5_A. (B) The structure of 5-phosphoribose alpha-1-diphosphate (1PP-R5P). This is cleaved to leave ribose (1,5)-bisphosphate and P_i_. Figure prepared in chembiodraw [138].

The diphosphoinositol polyphosphate phosphohydrolases catalyze the cleavage of the diphosphate moiety of diphosphoinositol polyphosphates to leave a monophosphate [111,112,115,116] (Fig. 2). They also catalyze the cleavage of dinucleoside oligophosphates [115–117] and 5-phosphoribose 1-diphosphate (1PP-R5P) [114] (Fig. 7), although the kinetic parameters for this latter compound suggest that this reaction is unlikely to occur to any significant extent *in vivo*. Measured kinetic parameters for these reactions are shown in Table 3. Most of the kinetic data are more than 10 years old [112,114–117] and, where two different laboratories have assayed the same enzyme with the same substrate, there has been an unfortunate divergence between the measured kinetic parameters. This needed to be addressed (i.e. there was a need for agreed procedures for the purification and preparation of the enzyme and substrate and a standardized assay) if progress was to be made in the study of the kinetics of these enzymes. There was also a noticeable and unfortunate lack of kinetic data regarding the hydrolysis of 1PP-IP_5_ and 1,5PP_2_-IP_4_. However, a study that addresses these issues has been published [118]. The study also confirms the finding made with the TNP inhibition of the IP6Ks [55] in that the synthesis of 1,5PP_2_-IP_4_ from IP_6_ occurs predominantly via 5PP-IP_5_ rather than via 1PP-IP_5_ (Fig. 2) and, conversely, the dephosphorylation of 1,5PP_2_-IP_4_ occurs preferentially via 1PP-IP_5_ [118]. Both DIPP-3 enzymes have an absolute requirement for divalent cations (with manganese being favoured) [116], as does DIPP-1 [111]. Given the high sequence homology of all the DIPP enzymes, they are all likely to require divalent cations for activity, although the role of the cations is unknown: they may play a role in neutralizing the negative charges of the substrate phosphates or may have a more direct role in the catalytic act. The DIPP enzymes are inhibited by fluoride [2,42,43].

**Table 3:** Kinetic parameters for the cleavage of pyrophosphate bonds. The data were taken from the references cited but converted into uniform units

Enzyme	Substrate	*K*_M_ (μm)	*k*_cat_ (s^−1^)	*k*_cat_/*K*_M_ (m^−1^·s^−1^ × 10^−3^)	Reference
DIPP-1	1PP-IP_5_	0.042	1.10	26190	[118]
	5PP-IP_5_	0.0042	0.20	47619	[117]
		0.052	0.13	2500	[118]
	1,5PP-IP_4_	0.085	0.10	1176	[118]
	Ap_6_A	5.9	0.50	85	[117]
	Ap_5_A	7.7	0.42	55	[117]
	1PP-R5P	380	1.00	2.6	[114]
DIPP-2α	1PP-IP_5_	0.060	0.05	833	[118]
	5PP-IP_5_	0.0042	0.15	35714	[112]
		0.035	0.07	2000	[118]
	1,5PP_2_-IP_4_	0.055	0.0024	44	[118]
DIPP-2β	1PP-IP_5_	0.070	0.017	243	[118]
	5PP-IP_5_	0.0048	0.03	6250	[112]
		0.040	0.003	75	[118]
	1,5PP_2_-IP_4_	0.042	0.0016	38	[118]
DIPP-3α	1PP-IP_5_	0.104	0.23	2212	[118]
	5PP-IP_5_	0.088	0.16	1818	[115]
		1.3	0.14	108	[116]
		0.146	0.04	274	[118]
	1,5PP_2_-IP_4_	–	–	180	[115]
		0.126	0.022	175	[118]
	Ap_6_A	33	0.58	17.6	[115]
		19	0.20	10.5	[116]
	Ap_5_A	50	0.80	16.0	[116]
DIPP-3β	1PP-IP_5_	0.073	0.08	1096	[118]
	5PP-IP_5_	0.053	0.22	4151	[115]
		4	0.20	50	[116]
		0.063	0.0088	140	[118]
	1,5PP_2_-IP_4_	–	–	490	[115]
		0.078	0.0037	47	[118]
	Ap_6_A	43	0.95	22.1	[115]
		13	0.17	13.1	[116]
	Ap_5_A	37	0.40	10.8	[116]

There are two crystal structures of human DIPP-1 with IP_6_ (a reaction product) bound (2FVV and 2Q9P) [119] and one of the human DIPP-3α apoenzyme (3MCF; unpublished). Also available is a structure of the murine DIPP-2 apoenzyme (2DUK; unpublished). The sequence of this protein (UniProtKB ID Q8R2U6) is highly homologous to that of human DIPP-2α, lacking the first methionine of the human enzyme and differing in just six other residues. The structural biology of DIPP has been reviewed [25].

**Fig. 8:**
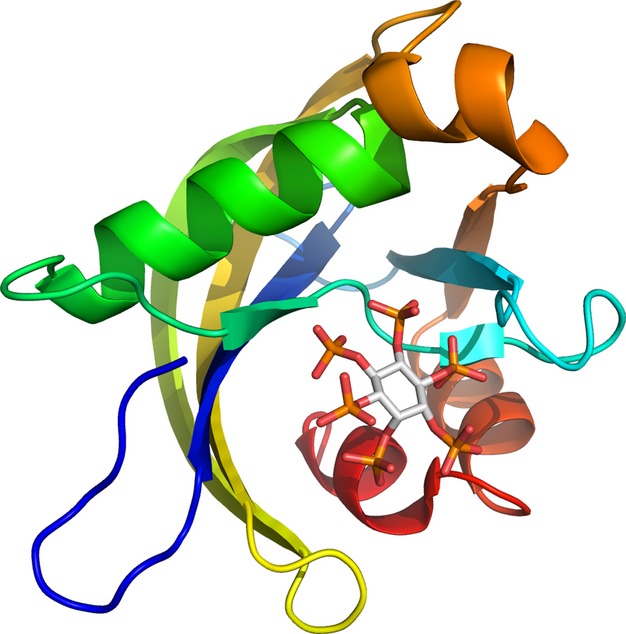
A cartoon showing the structure of DIPP-1. The protein, residues R10-E141, is coloured blue at the N-terminus through cyan, turquoise, green, yellow and orange to red at the C-terminus. IP_6_ with grey carbon atoms is shown in the substrate binding site. Taken from the 2Q9P structure [119]. Image prepared in pymol [139].

These structures have two β-sheets flanked by short helices (Fig. 8). The active site is formed largely by a mutated Nudix motif (residues G50-V73 in DIPP-1), which typically has the general form Gx_5_Ex_5_[UA]xREx_2_EExGU (where U represents an aliphatic, hydrophobic residue, and x is any residue) [120] but which, in the DIPP enzymes, has an extra residue inserted between the conserved N-terminal glycine and the conserved glutamate (Gx_6_E rather than Gx_5_E). This insertion changes the loop-helix-loop fold typical of the Nudix motif to strand-loop-helix, which is stabilized by a tight association between the first three residues of the Nudix motif and a neighbouring β-strand.

**Fig. 9:**
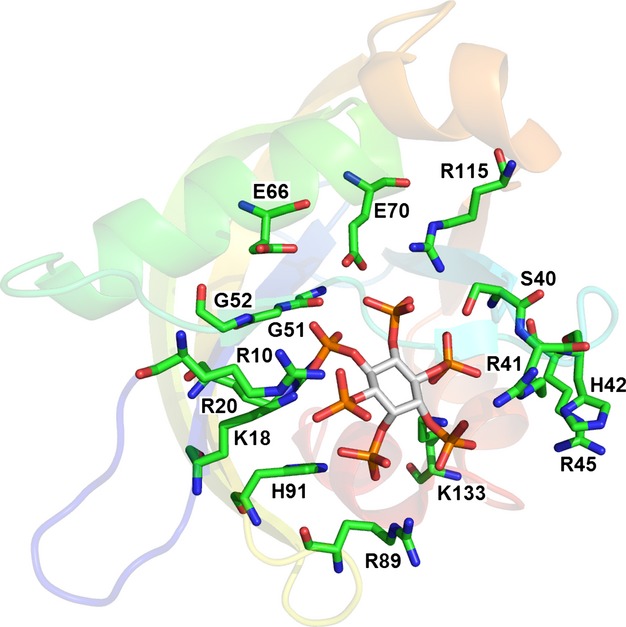
The residues forming the substrate binding site of DIPP-1. Taken from the 2Q9P structure [119]. Image prepared in pymol [139].

Although the Nudix motif forms the basis of the substrate binding site, some residues involved in binding or the catalytic act that are outside this motif have been identified in a detailed mutational study [121]. In DIPP-1, the G50A, G50V, G51A, G52A, G52V, E66Q, G72A, G75A, G78V and G82A mutations all cause a loss of function [121], as does the E70Q mutation [111]. The G78A mutation has no effect but the F84Y and H91L mutations cause a large decrease in Ap_6_A and 5PP-IP_4_ hydrolysis, with little effect on 5PP-IP_5_ hydrolysis [121]. Unsurprisingly, many of the residues forming the substrate binding site are, or can be, positively-charged to counter the negative charge of the substrate (six arginines, two lysines and two histidines) (Fig. 9). Based on the DIPP-1 structure with the reaction product, a reaction mechanism has been proposed [119], although the validity of this may be questionable because (a) two conformations of the product were observed in the binding site and the proposed mechanism was based on just one of these and (b) it was incorrectly assumed that the substrate was d-(3,5)-bisdiphospho-*myo*-inositol (1,2,4,6)-tetrakisphosphate (3,5PP_2_-IP_4_) rather than 1,5PP_2_-IP_4_.

Signal transduction through the ERK1/2 pathway is negatively regulated by murine DIPP-1 [122]. This regulation is observed even with a catalytically dead mutant, suggesting that DIPP-1, possibly in conjunction with an adaptor protein, binds to an enzyme on the ERK1/2 pathway [122].

In rats, levels of DIPP-2 mRNA increase in the frontal cortex of the brain upon prolonged dosing with lithium [123]. Because lithium has a role to play in the treatment of various psychiatric disorders, this suggests that diphosphoinositol polyphosphates themselves play a role in these disorders.

In the IntAct database (http://www.ebi.ac.uk/intact/) [67], DIPP-1 is identified as having interactions with (a) the GTPase Ras-related protein Rab-17 (UniProtKB ID Q9H0T7; gene name *RAB17*) that plays a role in membrane trafficking and exocytosis [124,125]; (b) the ankyrin repeat and SOCS box protein 13 (UniProtKB ID Q8WXK3; gene name *ASB13*) that may play a role in the ubiquitination and proteasomal degradation of target proteins [126]; (c) the 60S ribosomal protein L8 (UniProtKB ID P62917; gene name *RPL8*); (d) the 60S acidic ribosomal protein P1 (UniProtKB ID P05386; gene name *RPLP1*); (e) transcription factor SOX-30 (UniProtKB ID O94993; gene name *SOX30*); and (f) IQ calmodulin-binding motif-containing protein 1 (nephrocystin-5; UniProtKB ID Q15051; gene name *IQCB1*), which is probably involved in ciliogenesis, and mutations of which result in renal and retinal disease [127]. DIPP-3α is identified as interacting with Ras association domain-containing protein 1 (UniProtKB ID Q9NS23; gene name *RASSF1*), which is a potential tumour suppressor that regulates aspects of cell cycle progression and apoptosis [128]. DIPP-3β is identified as interacting with tumour necrosis factor receptor-associated factor 6 (UniProtKB ID Q9Y4K3; gene name *TRAF6*), which is a ubiquitin ligase [129].

## Discussion

Many roles have been attributed to the diphosphoinositol polyphosphates [17–25]. With more research, further roles are likely to be discovered. The activity of two types of kinase and a family of phosphohydrolases determines their turnover and concentration in cells. More work is needed to discover how the activity of these enzymes is regulated. As discussed above, and as shown in Table 4, the diphosphoinositol polyphosphate-metabolizing enzymes are capable of interacting with several different proteins with a range of functions. Whether or not these interactions are mutually exclusive (e.g. does the binding of Rab-17 to DIPP-1 prevent the binding of nephrocystin-5?) remains unknown. Also largely unknown is the effect of partner protein binding on catalytic activity. HSP90 inhibits the catalytic activity of IP6K2 [88] but, for most of the partner proteins listed in Table 4, the effect of the partner protein binding on catalytic activity is unknown.

**Table 4:** The interactions of the diphosphoinositol polyphosphate metabolizing enzymes. For references to these interactions, see text

Enzyme	Partner	Function
IP6K1	Exosome component 7 protein	RNA degradation
	Brain calcium channel 1 protein	Migraine, epilepsy
	GRAB	Exocytosis
	GSK3	Glycogen synthesis, diabetes
IP6K2	TRAF2	Apoptosis, infection/inflammation
	HSP90	Apoptosis
	p53	Apoptosis
	Caesin kinase 2	Apoptosis
	Numb-binding protein 2	Protein subcellular location
	Phospholipid scramblase 1	Phospholipids in membranes, interferon response
PPIP5K2	Myc	Cancer
DIPP-1	Rab-17	Membrane trafficking, exocytosis
	Ankyrin repeat and SOCS box protein 13	Ubiquitination, proteasomal degradation
	60S ribosomal protein L8	Translation
	60S acidic ribosomal protein P1	Translation
	SOX-30	Transcription
	Nephrocystin-5	Ciliogenesis
DIPP-3α	Ras association domain-containing protein 1	Tumour suppressor, apoptosis
DIPP-3β	TRAF6	Apoptosis, infection/inflammation, ubiquitin ligase

Both IP6K2 and DIPP-3β interact with TRAF proteins, possibly suggesting some sort of reciprocal regulation in response to infection and inflammation: the activity of IP6K2 is increased in response to binding TRAF2 and so it may be desirable to reduce the activity of DIPP-3β (which binds to TRAF6), although it is not known whether this is the consequence of DIPP-3β binding to TRAF6. Similarly, IP6K2 and both DIPP-3 enzymes bind to proteins involved in the apoptotic process: if the consequences of these interactions can be determined, this may be another example of reciprocal regulation. Many of these interactions have been found in proteomics studies which, although useful for determining the existence of the interactions, do not reveal the consequences of the interactions. More examples of reciprocal regulation of the kinases and the phosphatases may yet be discovered.

It should be noted that the enzymes of diphosphoinositol polyphosphate metabolism are not the only inositol phosphate-metabolizing enzymes to have noncatalytic functions. Inositol trisphosphate 3-kinase C (UniProt ID Q96DU7), which catalyzes the transfer of a phosphate from ATP to 1d-*myo*-inositol (1,4,5)-trisphosphate to generate 1d-*myo*-inositol (1,3,4,5)-tetrakisphosphate, is reported to interact with serine/threonine-protein phosphatase 2B catalytic subunit γ isoform (UniProt ID P48454), which dephosphorylates proteins [130]. Why these two proteins should interact is unclear (i.e. it may be that this interaction enables the phosphatase to dephosphorylate the kinase) but both are activated by calcium/calmodulin, as is the inositol 1,4,5-trisphosphate receptor (a calcium channel), which might suggest a role in regulating the intracellular movement of calcium or in the cellular response to such movement [26]. Inositol polyphosphate 1-phosphatase (UniProt ID P49441) catalyzes the removal of the 1-phosphate from 1d-*myo*-inositol (1,4)-bisphosphate and is involved in signal transduction and the phosphatidylinositol signalling pathway. It is reported to interact with DNA ligase 1 (UniProt ID P18858) [131]: the consequences of the interaction are unknown. Inositol tetrakisphosphate 1-kinase (UniProt ID Q13572) phosphorylates a number of inositol polyphosphates at various positions and is reported to interact with TRAF2 [63].

Neither are these noncatalytic functions limited to inositol phosphate-metabolizing enzymes. Catalytically dead mutants of the glycolytic enzyme triosephosphate isomerase play a crucial role in the behaviour and longevity of *Drosophila* [132]. Choline kinase α is important for the survival of cancer cells: inhibition of the catalytic activity is not sufficient to kill cancer cells but inhibition of expression results in significant cell death through apoptosis [133]. This implies a role for choline kinase α in cancer cell survival that is independent of its catalytic activity. Many other kinases have functions beyond their catalytic activities, including roles in protein scaffolds, DNA binding, subcellular targeting and allosteric effects on other enzymes [134].

A reviewer of the present study referred to the noncatalytic activities of enzymes as ‘moonlighting’ functions. This implies that the catalytic activity is the main function of the protein, with any other activity being something extra that the protein has acquired over time and which is of lesser importance. Although this may well be the case, it is by no means certain: an interaction with another protein might have been the original function, with the catalytic activity being acquired only later. Given the complexity of cells and signalling pathways, the multiple functions and interactions of many proteins, and the redundant systems in cells and pathways [135–137], it is probably difficult to identify definitively a single original function in a multifunctional protein.

The diphosphoinositol polyphosphates undergo rapid turnover: the inhibition of the DIPPs by fluoride results in a rapid accumulation of diphosphoinositol polyphosphates [2,42,43]. This turnover will be influenced by the subcellular localization and concentration of the enzymes and the substrates, the binding affinity of the enzymes for the substrates, and the presence of enzyme activators and inhibitors (which may include the proteins listed in Table 4). All the diphosphoinositol polyphosphate-metabolizing enzymes can be found in the cytoplasm or cytosol [10,54,81,82,95,97,115], with IP6K1 and IP6K2 also found in the nucleus [54,82]. PPIP5K1 has been found associated with the plasma membrane [16,99] and IP6K2 has been detected in mitochondria, although only during apoptosis [14]. The kinetic parameters for the three classes of enzyme are shown in Tables 1–3. The binding affinity of the DIPPs for the product of the IP6K- and PPIP5K-catalyzed reactions would suggest that there should be very little of the DIPP substrates detectable in the cell. The fact that the substrates can be detected suggests one or more of: (a) the concentration of the DIPPs is very low compared to the concentration of the IP6Ks and PPIP5Ks; (b) the DIPPs are in a separate subcellular compartment to the IP6Ks and PPIP5Ks; (c) the presence of intracellular inhibitors of DIPPs and/or activators of IP6Ks and PPIP5Ks reduces the relevance of the *in vitro* measurements of the activity of the purified enzymes to the *in vivo* situation; (d) phosphorylation (all these enzymes can be phosphorylated) may change the catalytic activity; or (e) the substrates are not accessible to the DIPPs because they are bound to other proteins or biomolecules.

The enzymes discussed herein have multiple functions and interactions: continuing research is likely to discover more of both. For a fuller understanding of these enzymes, many questions remain to be answered. How is their expression regulated? What is the concentration of the enzymes within cells? What is the subcellular localization of the enzymes? How, if at all, is this influenced by phosphorylation and/or interaction with partner proteins? Can the enzymes form ternary (or higher order) complexes with their interaction partners? What is the effect on catalytic activity of phosphorylation and/or interaction with partner proteins? What are the consequences of the interactions between proteins on cell and pathway function and behaviour? The answers will undoubtedly raise more questions relating to these still relatively enigmatic diphosphoinositol polyphosphates.

## Abbreviations

CK2: casein kinase-2
DIPP: diphosphoinositol polyphosphate phosphohydrolase
GSK: glycogen synthase kinase 3
IFN: interferon
IP_5_: *myo*-inositol (1,3,4,5,6)-pentakisphosphate
IP_6_: *myo*-inositol (1,2,3,4,5,6)-hexakisphosphate
IP6K: inositol hexakisphosphate kinase
5PA-IP_5_: 5-phosphonoacetate-*myo*-inositol (1,2,3,4,6)-pentakisphosphate
PBD: phosphoinositide binding domain
PH: pleckstrin homology
5PP-IP_4_: 5-diphospho-*myo*-inositol (1,3,4,6)-tetrakisphosphate
1,5PP_2_-IP_3_: 1,5-bisdiphospho-*myo*-inositol (3,4,6)-trisphosphate
1,5PP_2_-IP_4_: d-(1,5)-bisdiphospho-*myo*-inositol (2,3,4,6)-tetrakisphosphate
3,5PP_2_-IP_4_: d-(3,5)-bisdiphospho-*myo*-inositol (1,2,4,6)-tetrakisphosphate
1PP-IP_5_: d-1-diphospho-*myo*-inositol (2,3,4,5,6)-pentakisphosphate
5PP-IP_5_: 5-diphospho-*myo*-inositol (1,2,3,4,6)-pentakisphosphate
1PP-R5P: 5-phosphoribose 1-diphosphate
PPIP5K: inositol hexakisphosphate and diphosphoinositol-pentakisphosphate kinase
PtdIns(3,4,5)P_3_: phosphatidylinositol (3,4,5)-trisphosphate
TNP: *N*2-(*m*-(trifluoromethyl)benzyl) *N*6-(*p*-nitrobenzyl)purine
TRAF: tumour necrosis factor receptor-associated factor
